# Microstructural Investigation and On-Site Repair of Thin Pd-Ag Alloy Membranes

**DOI:** 10.3390/membranes10120384

**Published:** 2020-11-30

**Authors:** Yuyu Ma, Chunhua Tang, Feng Bao, Wei Shao, Tianying Xu, Hui Li, Hengyong Xu

**Affiliations:** 1 Dalian Institute of Chemical Physics (DICP), Chinese Academy of Sciences, Dalian 116023, China; mayy2019@dicp.ac.cn (Y.M.); chtang@dicp.ac.cn (C.T.); baofeng@dicp.ac.cn (F.B.); shaowei@dicp.ac.cn (W.S.); xutianying@dicp.ac.cn (T.X.); xuhy@dicp.ac.cn (H.X.); 2 University of Chinese Academy of Sciences, Beijing 100049, China

**Keywords:** Pd-Ag membranes, ELP and EP, uniform alloy, heating/cooling cycles, on-site repair

## Abstract

Pd membranes act in an important role in H2 purification and H2 production in membrane reactors. Pd-Ag alloy membranes fabricated by consecutive electroless- and electroplating process on alumina tubes exhibited good stability under stringent heating/cooling cycles at a ramp rate of 10 K/min, imitating practical fast initiation or emergency shutdown conditions. Bilayer Pd-Ag membranes can form dense and uniform alloy after thermal treatment for 24 h at 823 K under H2 atmosphere, despite a porous structure due to the development of liquid-like properties above Tamman temperature to enforce the migrativity. On the contrary, alloying under N2 atmosphere resulted in a Pd-enriched layer. This led to a lower H2 flux but superior thermal stability compared to that alloying under H2 atmosphere. The trilayer approach of electroless-plated Pd, electro-polated Ag and electroless-plated Pd is not suitable to achieve homogeneous Pd-Ag alloys, which, on the other hand, presented the occurrence of a small gap between top Pd layer and middle Ag layer, probably due to insufficient wetting during plating process. An on-site repair treatment in analogous to MOCVD (Metal-organic Chemical Vapor Deposition) process was first proposed to extend the lifetime of Pd-Ag membrane, i.e., by vaporizing, and subsequent decomposition of Ag(OOCC2F5) powders to “preferentially” block the pinholes under vacuum and at working temperature of ca. 473–673 K, which effectively reduced the N2 flux by 57.4% compared to the initial value. The H2 flux, however, declined by 16.7% due to carbon deposition on the membrane surface, which requires further investigation. This approach shows some potential for on-site repair without disassembly or cooling to room temperature.

## 1. Introduction

Pd and its alloy membranes have attracted extensive attention for their application in hydrogen purification and hydrogen production in membrane reactors, owing to their extraordinary hydrogen permeability and selectivity [1,2,3,4]. The formation of leaks or delamination is, however, often reported for thin Pd composite membranes during thermal treatment and/or hydrogen absorption/desorption cycles [5,6,7], for example, due to grain growth, interdiffusion, the difference in thermal expansion coefficients or inefficient adhesion strength between Pd metal layer and support. In addition, significant extension and shrinking occur during the hydrogen absorption/desorption process, as reported for self-supported finger-type Pd-Ag tubes [8,9], which may also occur in case of thin supported Pd membranes. Different approaches have been proposed in the literature to enhance the durability of composite membranes, such as porous support modification [10], internal coating of Pd layer on porous support to constrain its thermal expansion [11] and improving anchorage of metal layer to porous support by modification with a zeolite layer [12]. There is also a method to achieve high and stable hydrogen permeability of palladium-silver films at low temperatures by coating the surface [13]. The formation of Pd-Ag alloy can effectively increase hydrogen permeability of Pd membranes [14], and simultaneously improve the durability and resistance to hydrogen embrittlement at temperatures below 573 K [15], which focused great attention from both the academia and industry [16,17,18,19]. The content of silver determines the critical temperature of the α-β transition, as well as the hydrogen permeability of supported Pd-Ag alloy membranes [2,19], whereas it is not easy to control the Ag content, microstructure and morphology during the fabrication process. For instance, the Ag films prepared by the electroless-plating method exhibit dendritic morphology and highly nonuniform growth, and tend to grow in the vertical direction on Pd membrane surface, with poor lateral growth or pore coverage [4]. The magnetron sputtering method exhibits improved control in both composition and microstructure through multicomponent targets, where ultra-thin, uniform and dense Pd-Ag membranes can be obtained [14,20,21] (. However, the equipment of magnetron sputtering is relatively expensive and requires a strict fabrication procedure, while membrane geometry is often limited to be a flat type. The electroless co-deposition method was developed to obtain a uniform distribution of Pd-Ag alloy membranes [22], but much exploration is required to determine the appropriate preparation conditions. Particularly, it is challenging to obtain target composition with this approach [2]. The alloying process of Pd-Ag membranes following sequential electroless deposition often requires hundreds of hours [15,23]. In most literature studies, the alloying process following electroless plating is completed under hydrogen atmosphere [3,4,11,15,19,24]. Jia et al. [25] observed the formation of cavities on the PdCuAu membrane when alloying under hydrogen atmosphere, which became larger during the following testing in H_2_ at 873 K. In contrast, there was no cavity formation observed with a dense morphological feature when alloying under N_2_ atmosphere at 1073 K.

It is important to identify appropriate composition, microstructure and morphology to achieve required durability and reliability particularly when undergoing fast initiation or emergency shutdown scenarios, e.g., fuel cell application or during power-off accidents.

Moreover, palladium membranes are subject to defects during preparation or application processes, and there are a number of membrane repair methods available. For example, Zeng et al. [26] performed defect repair by adding metal ions and reducing agents on both sides of the palladium film, slightly pressurizing it to cause a gradient of reducing agent diffusion through the defect to the palladium solution. Lu et al. [27] followed metal bonding to repair the palladium film. Lundin et al. [28] sealed the defects on the palladium film with glass powder. Zheng et al. [29] used homemade “modified” liquid–liquid displacement porometry (MLLDP) to characterize the defect size, followed by the repair of defects in the palladium composite film by filling with high-temperature-resistant silicate gel (HTRSG) composite ceramic particles. However, the current repair methods are mostly offline. In this study, a bilayer or trilayer electroless-plating Pd and electroplating Ag membrane was fabricated and alloyed under different atmospheres. The microstructure, deposition morphology and topological features were investigated in detail, which were correlated with the following stringent stability study imitating practical fast initiation or shutdown scenarios. In addition, an onsite repair treatment was first proposed by vaporizing and the subsequent decomposition of Ag(OOCC_2_F_5_) powders at working temperatures to “preferentially” block the pinholes under vacuum conditions without membrane disassembly.

## 2. Experiments

### 2.1. Fabrication of Pd-Ag Alloy Membranes

The fabrication of Pd-Ag/Al_2_O_3_ composite membranes consisted of several steps: (i) The porous alumina tubes with an average pore size of 100 nm and a length of 62–136 mm were cleaned sequentially by ethanol, 4% aqueous KOH solution and deionized water, which were denoted as PA-1 and PA-2 and PAP-1 and PAP-2 (They were labeled with the plating sequence of the Pd and Ag layers, i.e., PA (two layers in the sequence: electroless-plated Pd and then electroplated Ag) and PAP (three layers in the sequence: electroless-plated Pd, electroplated Ag and electroless-plated Pd).). (ii) A ceramic suspension was prepared containing alumina powders (200 nm), polyvinyl alcohol (PVA) and polyethylene glycol (PEG), which was then used to modify the external surface of the Al_2_O_3_ substrate via impregnation and a drying process (at 423 K, 4 h). (iii) A thin Pd layer was prepared through the seeding and the electroless-plating method (ELP) described previously [30], onto which a thin Ag layer was deposited through the electroplating technique (EP; the plating solution was purchased from Tian Yue Chemical, Shenzhen, China, T: 231 K). In the case of PAP-1 and PAP-2, a second Pd layer was manufactured on top of the Ag layer via the electroless-plating method. (iv) The multilayer Pd-Ag membranes were then annealed at 823 K for 24 h under H_2_ or N_2_ atmosphere in order to form homogeneous alloys. The performance data of the Pd-Ag alloy membranes investigated in this study are listed in Table 1.

### 2.2. Membrane Characterization

The surface and cross-section of Pd-Ag membranes were examined before and after the alloying process by scanning electron microscopy (SEM, JSM-7800F, Tokyo, Japan) equipped with energy-dispersive X-ray spectroscopy (EDS). The crystal structure of the alloy membranes was determined by X-ray diffraction (XRD, X’pert Pro-1, Almelo, Netherlands) using Cu Ka radiation with the voltage set at 40 kV and current at 40 mA.

### 2.3. Gas Permeation Measurement

The Pd-Ag alloy membranes were sealed and mounted into a stainless-steel cell shell using graphite gaskets and then placed inside a furnace that featured a programmable temperature controller, as described previously [31]. The feed gas flow and pressures were set with a mass flow controller in the feed and a back-pressure valve in the retentate line, respectively, while the actual feed pressure was measured with a pressure gauge. Note that the permeation side was kept at atmospheric pressure without using sweep gas during permeation measurements. Both the feed and permeate sides were thoroughly swept with N_2_ before leak rate measurements, while single gas flux was measured with a soap bubble flow meter and the larger ones (>1000 mL min^−1^) with a THZM8 electronic soap film flow meter (Wuhan Tianhong Instrument Co., Ltd., Wuhan, China). Permeation rates were determined between 523 K and 723 K at pressure differences of up to 400 kPa, feeding H_2_ or N_2_ to the shell side. The activation energy for H_2_ permeation (*E*_act_) was determined by the linear least-squares fitting of Arrhenius plots of the H_2_ fluxes (*J*_H2_) measured between 523 K and 723 K at a 100-kPa pressure difference. Besides, we carried out H_2_/N_2_ exchange cycles at 423–623 K under atmospheric pressure, where H_2_ and N_2_ fluxes were recorded at ΔP = 100 kPa during fast cycling experiments at a ramp rate up to 10 K/min to monitor the stability and integrity of Pd-Ag alloy membranes.

### 2.4. Onsite Repair of Pd-Ag Composite Membranes

**Figure 1:**
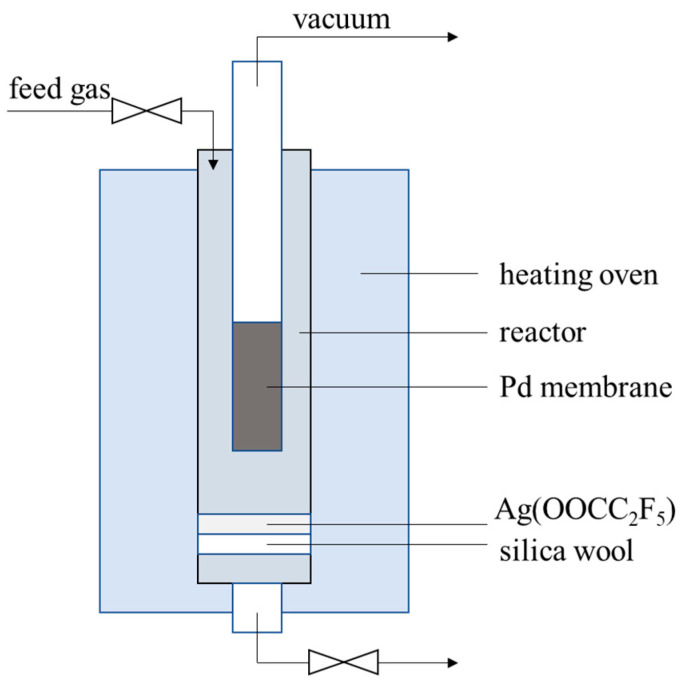
The illustration of the membrane cell for onsite repair treatment.

The membrane cell for onsite repair treatment was illustrated in Figure 1. About 0.5-g Ag(OOCC_2_F_5_) was placed beneath the membrane tube in the cell, which was heated up together with the membranes and vaporized to block the pinholes. Note that a vacuum was applied from inside of the membrane (200 Pa), while the temperature was increased from room temperature to 673 K at a ramp rate of 2 K/min, followed by keeping at 673 K for 1 h; after reparation, the membrane was cooled down in N_2_ atmosphere at a flow rate of 150 mL/min. The cycling test was also carried out after the onsite repair treatment, as described in Section 2.3.

## 3. Results and Discussion

### 3.1. Characterization of Pd-Ag Alloy Membranes

The four Pd-Ag alloy membranes investigated in this study are depicted in Table 1, denoted as PA-1, PA-2, PAP-1 and PAP-2, respectively. According to weight gain after Pd plating and electric quantity after Ag plating, the thicknesses of these Pd-Ag alloy membranes were calculated as ca. 6.14–6.33 μm, which differed slightly from those analyzed by cross-section SEM images. As to bilayer PA-1 and PA-2 prepared via electroless plating (ELP) Pd layer/electroplating (EP) Ag layer, the silver content was around 33.23–46.17 wt.% by EDS analysis, which was significantly reduced to 8.65–10.42 wt.% for trilayer PAP-1 and PAP-2 with a second ELP Pd layer. All elements analyzed in the table are shown in Tables S1–S4. The silver content analyzed by XRD according to Vegard’s fit closely to the values of the EDS analysis. For PAP-1 and PAP-2, the gravimetric measurement suggested 17.05–24.38 wt.% Ag for the entire membrane, but the EDS was 8.65–10.42 wt.% Ag. Thus, the surface appeared Pd-enriched. Furthermore, the XRD then gave 9.85–13 wt.% Ag. This means XRD gave a value closer to the bulk composition of the membrane, which is also very reasonable, because XRD has a deeper penetration than EDS. Thus, for samples with surface enrichment of one alloy, the XRD analysis is expected to be closer to the bulk composition than the EDS.

**Figure 2:**
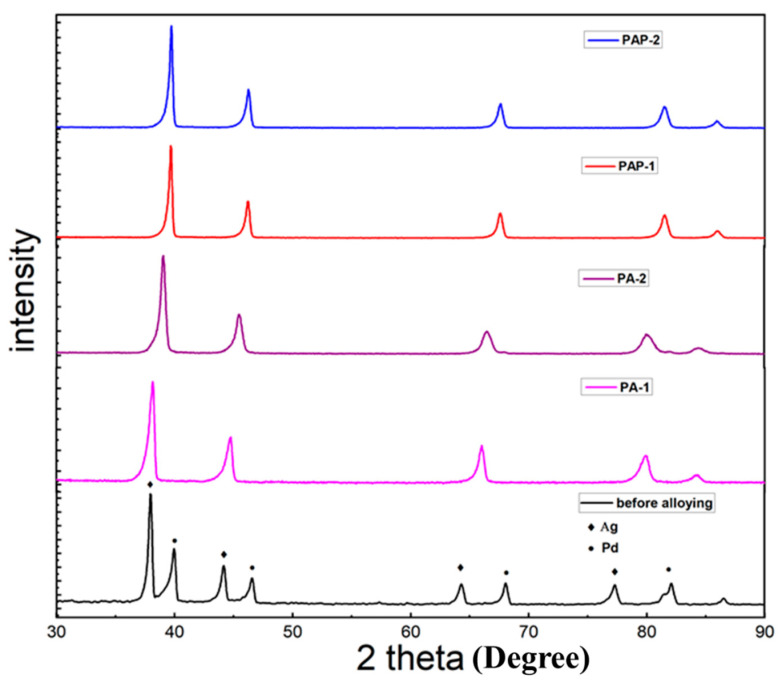
X-ray diffraction (XRD) patterns of PA-1 before alloying; PA-1, PA-2, PAP-1 and PAP-2 after annealing treatment at 823 K for 24 h.

This study is dedicated to investigating the influence of Ag deposition conditions on alloying behaviors, with the Ag layer deposited either above or below Pd layer. Figure 2 exhibits the XRD pattern of the four membranes investigated, i.e., PA-1, PA-2, PAP-1 and PAP-2, after annealing treatment at 823 K for 24 h, where only Pd-Ag alloy peaks can be observed after alloying. It is supposed that H_2_ treatment at high temperatures above 550 °C can significantly promote the migration and diffusion of Pd atoms, as previously reported with in-situ SEM (Zhu, 2017 #31). There are different opinions in the literature that the position of the silver film (below or above the Pd layer) will lead to different alloying effects. For example, Ag atoms will preferentially segregate towards the film surface when deposited on top of Pd layers to reduce the surface energy, leading to the incomplete formation of Pd-Ag alloys [32]. On the other hand, the chemical adsorption of hydrogen to palladium will lead to adsorbate-induced segregation when depositing Ag layer on Pd layers, thus beneficial to Pd-Ag alloying [15].

**Figure 3:**
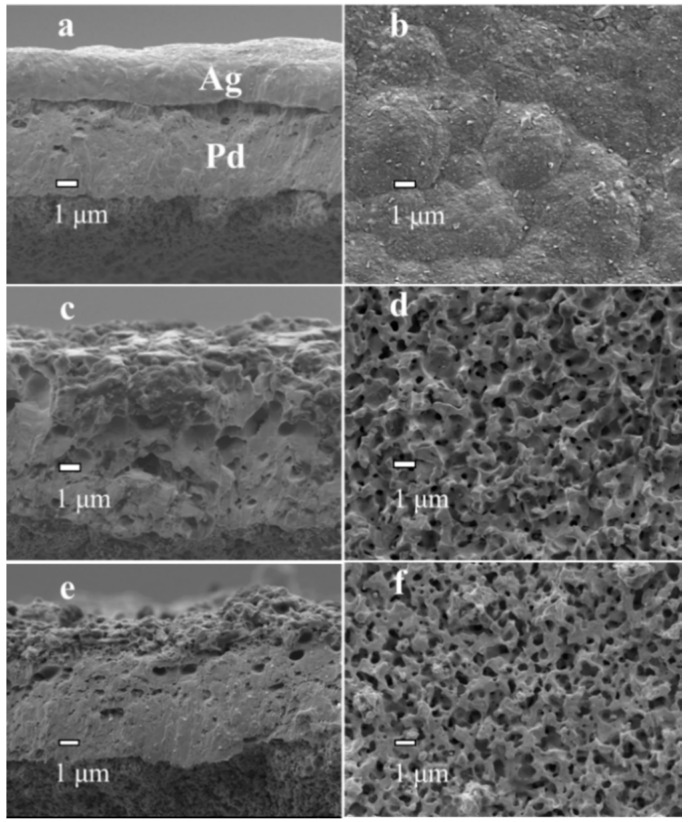
SEM images of Pd-Ag alloy layers: (**a**,**b**) PA-1 before alloying, (**c**,**d**) PA-1 after alloying under hydrogen at 823 K for 24 h and (**e**,**f**) PA-2 after alloying under nitrogen at 823 K for 24 h.

**Figure 4:**
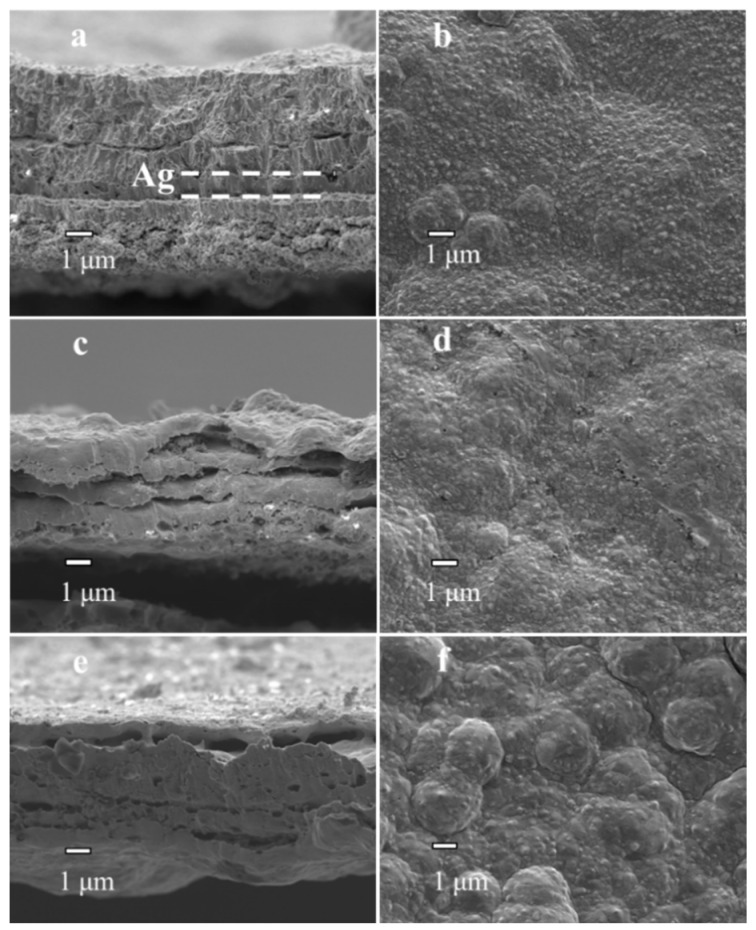
SEM images of Pd-Ag alloy layers: (**a**,**b**) PAP-1 before alloying, (**c**,**d**) PAP-1 after alloying under hydrogen at 823 K for 24 h and (**e**,**f**) PAP-2 after alloying under nitrogen at 823 K for 24 h.

PA-1, PA-2, PAP-1 and PAP-2 with the Ag layer deposited either above or below the Pd layer were further investigated by SEM analysis (Figure 3 and Figure 4). The cross-sectional images of PA-1 and PAP-1 as deposited (before annealing treatment at 823 K) are exhibited in Figure 3a and Figure 4a, respectively. There is a very distinctive boundary line between the Pd and Ag layers in both PA-1 and PAP-1. Obviously, the boundary line is more obvious for PA-1 than that of PAP-1, which became not significant after the alloying treatment for both membranes, as in Figure 3c and Figure 4c. Judging by the position in Figure 4c,e, the delamination for PAP-1 and PAP-2 mainly occurs between the top ELP Pd layer and EP Ag layer (middle layer), which probably originated from alloying process with partial segregation of the top Pd layer under thermal treatment and hydrogen absorption conditions. Nevertheless, the bilayer PA-1 membrane with EP Ag layer on top exhibited a uniform morphology after treatment at 823 K for 24 h, despite a porous structure of Pd-Ag alloy layer. This is ascribed to the development of liquid-like properties with enhanced migrativity following the high-temperature annealing treatment above the Tamman temperature of Ag. On the other hand, PA-1 and PA-2 possess a H_2_/N_2_ selectivity level of 3439 and 2403, respectively.

The top surface of PAP-1 and PAP-2 exhibit a polycrystalline structure with a low selectivity of 23 and 138, respectively. This is probably related to the fact that the bottom Pd layer dominates the permeation selectivity, while the EP Ag plating occurs at the position of the existing Pd metal due to low electric resistance, thus not capable of filling the voids or pinholes of the first ELP Pd layer. Therefore, it is challenging to improve the selectivity by EP plating [33,34]. It is noted in Table 1 that the activation energy of PAP-1 and PAP-2 is between 25.16 and 30.38 kJ/mol, significantly above that of PA-1 and PA-2 (6.81 and 14.06 kJ/mol, respectively). The values of activation energy in the literature range from 4 to 11 kJ/mol when the silver content is between 0 and 20 wt.% [35]. Therefore, the high activation energies of PAP-1 and PAP-2 may be ascribed to the small gap between different layers, as shown in Figure 4, and nonhomogeneity between different layers, which increased the diffusion resistance of the hydrogen atoms. This may also account for the difference in content results between gravimetric and EDS analyses in Table 1. The small gaps were ascribed to insufficient wetting of the middle Ag layer during electroless Pd plating.

**Figure 5:**
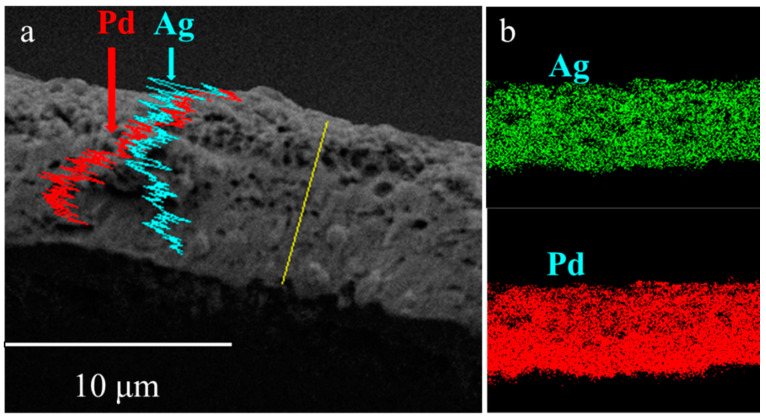
Cross-sectional X-ray spectroscopy (EDS) analysis of (**a**) PA-2 and (**b**) PA-1.

The EDS diagram of the PA-2 cross-section (Figure 5a) presented a nonuniform layer that Pd enriched at the bottom. The incomplete alloying was ascribed to a lack of annealing time under N_2_ atmosphere. Elemental mapping of PA-1 is shown in Figure 5b, which shows that PA-1 has a uniform alloy. A porous structure was also observed for PA-2 similar to PA-1, implying that the thermal treatment temperature rather than gas atmosphere dominated the morphology development during alloying process.

The Pd deposition in Figure 4f after alloying in N_2_ atmosphere appeared to be in the form of larger clusters, in contrast to the PAP-1 alloying in H_2_ atmosphere, as shown in Figure 4d. It has been observed with an in-situ SEM analysis that the H_2_ treatment exhibits a homogenization/diffusion effect along the whole membrane surface due to enhanced migrativity [36]. In this study, the N_2_ treatment resulted in the evolution of micro-hillocks analogous to active O_2_ or H_2_O species, which showed reactivity towards Pd metal [36].

### 3.2. Permeation and Stability Test

**Figure 6:**
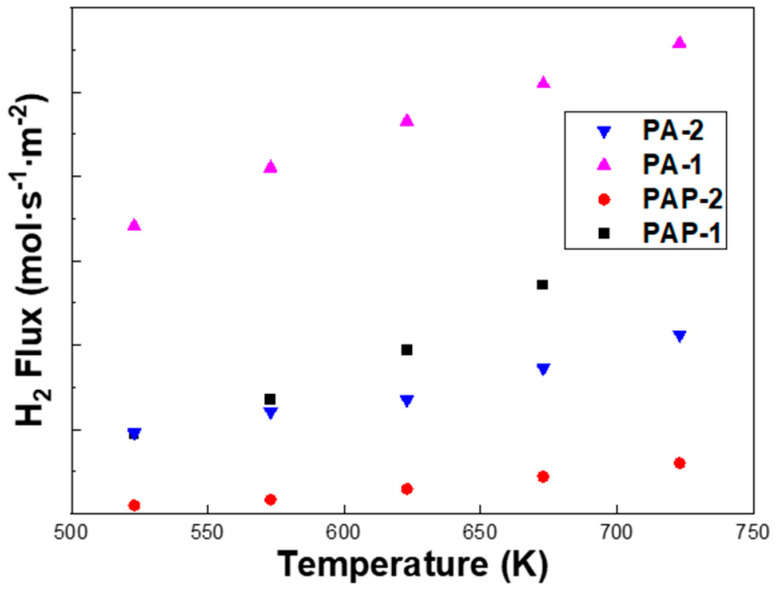
Temperature dependence of H_2_ permeation flux (Δ*P*_H2_ = 200 kPa) of PA-1, PA-2, PAP-1 and PAP-2.

**Figure 7:**
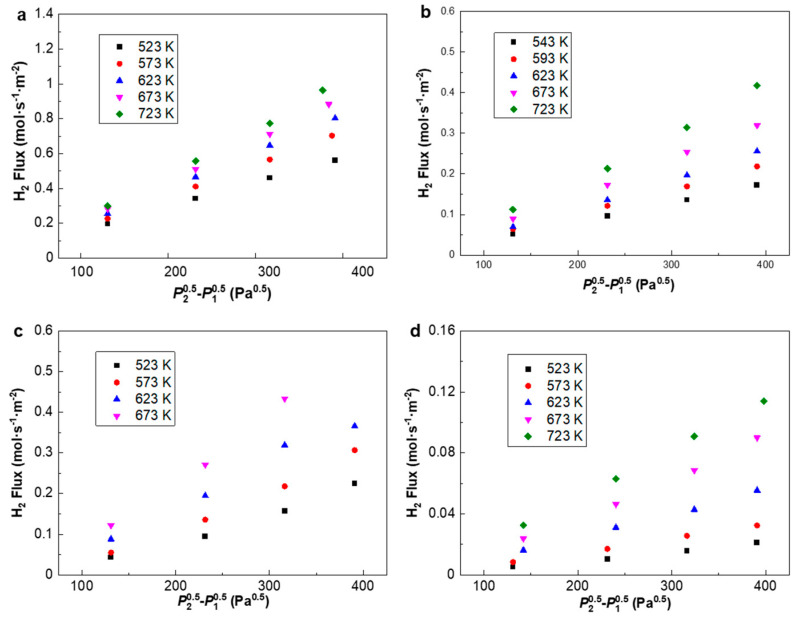
Hydrogen flux profile of (**a**) PA-1, (**b**) PA-2, (**c**) PAP-1 and (**d**) PAP-2 plotted against *P*_2_-*P*_1_ at 523 K–723 K. *P*_2_ and *P*_1_ indicate the feed and permeate pressures, respectively.

It can be seen from Figure 6 that the H_2_ flux of PA-1 is almost three times higher than that of PA-2, despite a slightly higher thickness from SEM images, i.e., 8.55 μm vs. 6.67 μm. Note that PA-2 exhibits a higher Ag content of 46.17 wt.% than the 33.23 wt.% of PA-1 according to the EDS analysis, which partly accounted for the lower H_2_ permeation flux of PA-2. The permeation flux was measured for PA-1, PA-2, PAP-1 and PAP-2 at 523–723 K, with a pressure differential of 1–4 bar (Figure 7). The fit of the pressure exponent *n* was calculated as 0.5, indicating that hydrogen diffusion in the bulk dominated the rate-limiting step in H_2_ permeation across these alloy membranes.

**Figure 8:**
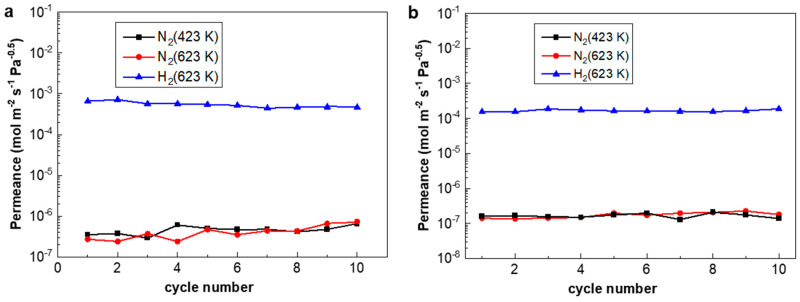
The performance of (**a**) PA-1 and (**b**) PA-2 during rapid heating/cooling cycles between 423 K and 623 K at a ramp rate of 10 K/min.

The H_2_/N_2_ thermal cycles were carried out between 423 K and 623 K at a ramp rate of 10 K/min, simulating fast initiation/shut-down in fuel cell applications or during power-off accidents. Figure 8 shows that both the H_2_ flux of PA-1 and PA-2 remained nearly unchanged, while the N_2_ flux of PA-1 increased slightly and that of PA-2 was relatively stable. Accordingly, the H_2_/N_2_ selectivity of PA-1 decreased from 3439 to 1050 after 10 fast temperature and gas cycles, while that of PA-2 showed a steady value before and after the cycling test. The performance of the PA-2 membrane alloyed under N_2_ atmosphere exhibited relatively stable behavior under stringent fast cycles than PA-1 alloyed under H_2_ atmosphere. Thermal stability is still a critical obstacle towards the application of thin Pd composite membranes. For example, severe elongation and contraction [8] exist in self-supported Pd-Ag tubes during hydrogen absorption/desorption cycles.

Chemical vapor deposition (CVD) is a common approach to fabricate thin Pd composite membranes, where one or several volatile palladium precursors are thermally decomposed on the surface of the substrate to obtain a metal film [37]. Here, an onsite membrane repair approach was inspired by the CVD process, where Ag(OOCC_2_F_5_) was chosen as the precursor for silver deposition [38], decomposing at ca. 473 K to block the defects in the form of Ag particles (Ag(OOCC_2_F_5_)→Ag + CO_2_ + CF_3_˙ + C_2_F_5_˙ + C_2_F_2_) [39]. Ag(OOC_2_F_5_) was chosen in this study as it was the precursor in the CVD process for silver deposition, and the deposition temperature of ca. 473 K was within the working range of Pd membranes. Other alternatives, such as Pd precursors for the CVD process with a proper working temperature, can also be available in order to block the membrane pinholes. During repair treatment, the Ag(OOCC_2_F_5_) particles were located beneath a Pd-Ag membrane tube inside the cell. Then, the membrane cell was heated up to 673 K while a vacuum level of 200 Pa was applied at the permeation side of the Pd-Ag membrane in order to preferentially deposit the decomposition products at the positions of the pinholes or defects.

**Figure 9:**
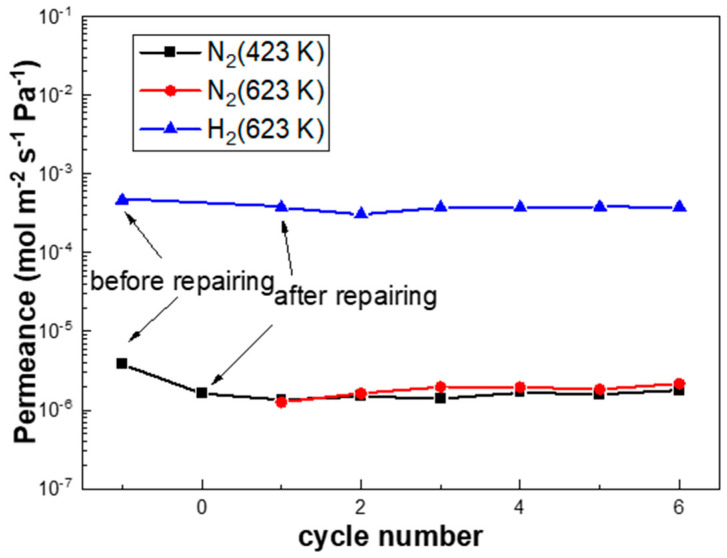
The performance of PA-1 after repairing during rapid heating/cooling cycles between 423 K and 623 K at a ramp rate of 10 K/min.

Figure 9 shows that the N_2_ leak rate of PA-1 declined by 57.4% after the repair treatment, which remained stable during the subsequent rapid temperature/gas cycles between 423 K and 623 K at a ramp rate of 10 K/min. This indicated good stability of the membranes with such an onsite repair treatment. Notably, the hydrogen permeation of the PA-1 membrane was decreased by 16.4%, possibly due to the formation of carbon species on the membrane surface according to the reaction formula (Ag(OOCC_2_F_5_)→Ag + CO_2_ + CF_3_˙ + C_2_F_5_˙ + C_2_F_2_). This approach shows some potential for onsite repair treatments at working temperatures (e.g., 523–673 K) without disassembly or cooling down to room temperature, which need to be further optimized.

**Figure 10:**
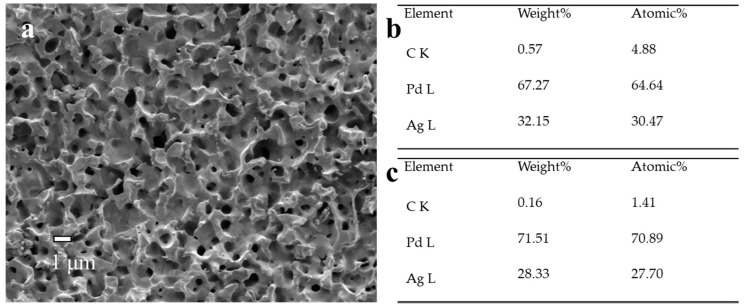
(**a**) SEM image, (**b**) surface EDS analysis and (**c**) cross-sectional EDS analysis of PA-1 after repair.

We carried out the SEM and EDS analyses of Pd membranes before and after reparation, and there was no significant difference in the surface morphologies observed (Figure 3d and Figure 10). The higher carbon content on the membrane surface after repair (0.57 wt.%) compared to that on the cross-section (0.16 wt.%) was directly related with the decomposition of the Ag(OOCC_2_F_5_) precursor, leading to a slight accumulation of carbon. Besides the membrane surface, the deposition was supposed to occur on the reactor walls, which might generate some Ag particles within the reactor. This is a disadvantage for this reparation approach.

## 4. Conclusions

This study investigates the microstructure, thermal stability and onsite repair of bilayer and trilayer Pd-Ag alloy membranes fabricated by sequential electroless- and electroplating processes on porous alumina tubes. Thin and uniform Pd-Ag alloy membranes can be obtained via the bilayer approach of electroless-plating Pd and electroplating Ag, followed by H_2_ treatment at 823 K at 24 h. In spite of a porous structure due to liquid-like properties developed above a Tamman temperature, the bilayer Pd-Ag alloy membranes exhibited good performance and stability under stringent temperature/gas cycles at a ramp rate of 10 K/min, imitating fast initiation/emergency shut-down scenarios, e.g., during power-off accidents. Alloying under N_2_ resulted in a Pd-enriched bottom layer, leading to a lower H_2_ flux but superior thermal stability compared to alloying in H_2_ atmosphere. The trilayer electroless deposition of the Pd layer onto the electroplated Ag layer was not suitable to achieve homogeneous Pd-Ag alloys presented in a small gap between the top Pd layer and middle Ag layer, probably due to insufficient wetting during the plating process. An onsite repair approach at a working temperature was first proposed, with Ag(OOCC_2_F_5_) powders vaporized and preferentially decomposed at the position of the defects under vacuum conditions. After the repair treatment, the N_2_ flux was reduced by 57.4% compared to the initial value, while the H_2_ flux declined by 16.7%, which was probably due to carbon deposition on the membrane surface. This novel approach exhibited potential for membrane repair in a working environment without disassembly or cooling down to room temperature.

## Figures and Tables

**Table 1:** Performance data of the Pd-Ag alloy membranes investigated in this study.

Sample	Method	d ^a^ μm	d ^b^ μm	Ag ^a^ wt.%	Ag ^b^ wt.%	Ag ^c^ wt.%	Ea kJ·mol^−1^	*F*_H2_^c^ × 10^4^ mol·s^−1^·m^−2^·Pa^−0.5^	α_H2/N2_^d^
PA-1	ELP_Pd_ + EP_Ag_	6.14 ± 0.6	8.55	30.80	33.23	36.40	6.81	8.927	3439
PA-2	ELP_Pd_ + EP_Ag_	6.14 ± 0.6	6.67	30.80	46.17	44.52	14.06	2.818	2403
PAP-1	ELP_Pd_ + EP_Ag_ + ELP_Pd_	6.33 ± 0.6	4.28	17.05	10.42	13.06	25.16	2.935	23
PAP-2	ELP_Pd_ + EP_Ag_ + ELP_Pd_	6.18 ± 0.6	5.32	24.38	8.65	9.85	30.38	0.753	138

^a^ Pd-layer thickness was calculated from weight gain after electroless plating, and Ag-layer thickness was calculated from the electric quantity. ^b^ SEM and X-ray spectroscopy (EDS) after alloying. ^c^ X-ray diffraction (XRD) after alloying. ^d^ 673 K and Δ*P* = 100 kPa. ELP: electroless-plating method and EP: electroplating.

## Supplementary Materials

The following are available online at https://www.mdpi.com/2077-0375/10/12/384/s1: Table S1: EDS analysis of Pd and Ag content of PA-1; Table S2: EDS analysis of Pd and Ag content of PA-2; Table S3: EDS analysis (cross-section and surface) of Pd and Ag content of PAP-1; Table S4: EDS analysis of Pd and Ag content of PAP-2 of PAP-2.
