# Supplementary material for: Microstructural Investigation and On-Site Repair of Thin Pd-Ag Alloy Membranes
Source: Membranes (Basel). 2020 Nov 30;10(12):384. doi: 10.3390/membranes10120384 (PMC7760571; doi:10.3390/membranes10120384)

Supporting information

Table S1 EDS analysis of Pd and Ag content of PA-1

Element	Weight%	Atomic%
Pd L	66.77	67.08
Ag L	33.23	32.92
Totals	100.00	

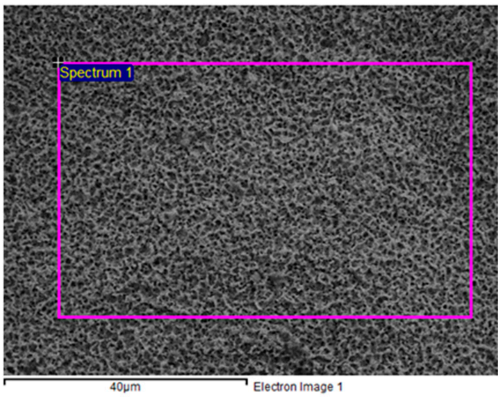

Table S2 EDS analysis of Pd and Ag content of PA-2

Element	Weight%	Atomic%
Pd L	53.83	54.17
Ag L	46.17	45.83
Totals	100.00	

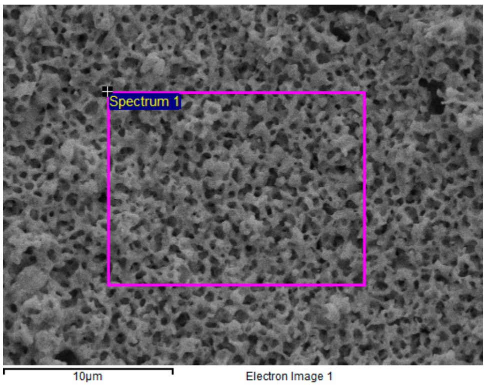

Table S3 EDS analysis (cross-section and surface) of Pd and Ag content of PAP-1

Element	Weight%	Atomic%
Pd L	89.90	90.03
Ag L	10.10	9.97
Totals	100.00	

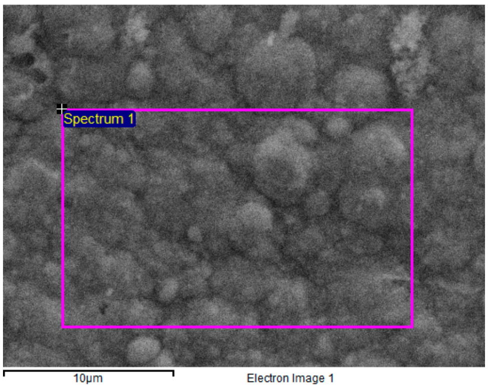

Element	Weight%	Atomic%
Pd L	89.58	89.71
Ag L	10.42	10.29
Totals	100.00	

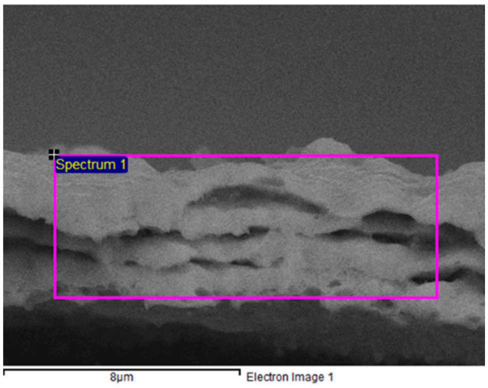

Table S4 EDS analysis of Pd and Ag content of PAP-2 of PAP-2

Element	Weight%	Atomic%
Pd L	91.35	91.46
Ag L	8.65	8.54
Totals	100.00	

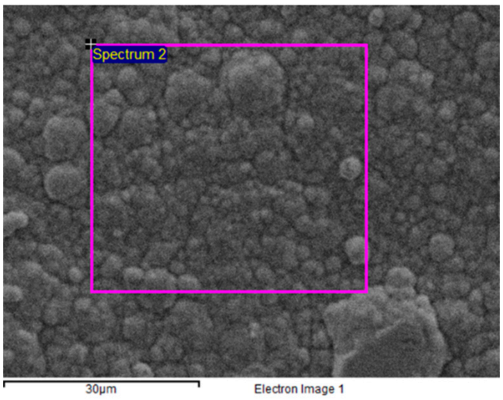

Supplement: Supplementary file 1 [file membranes-10-00384-s001.pdf]
